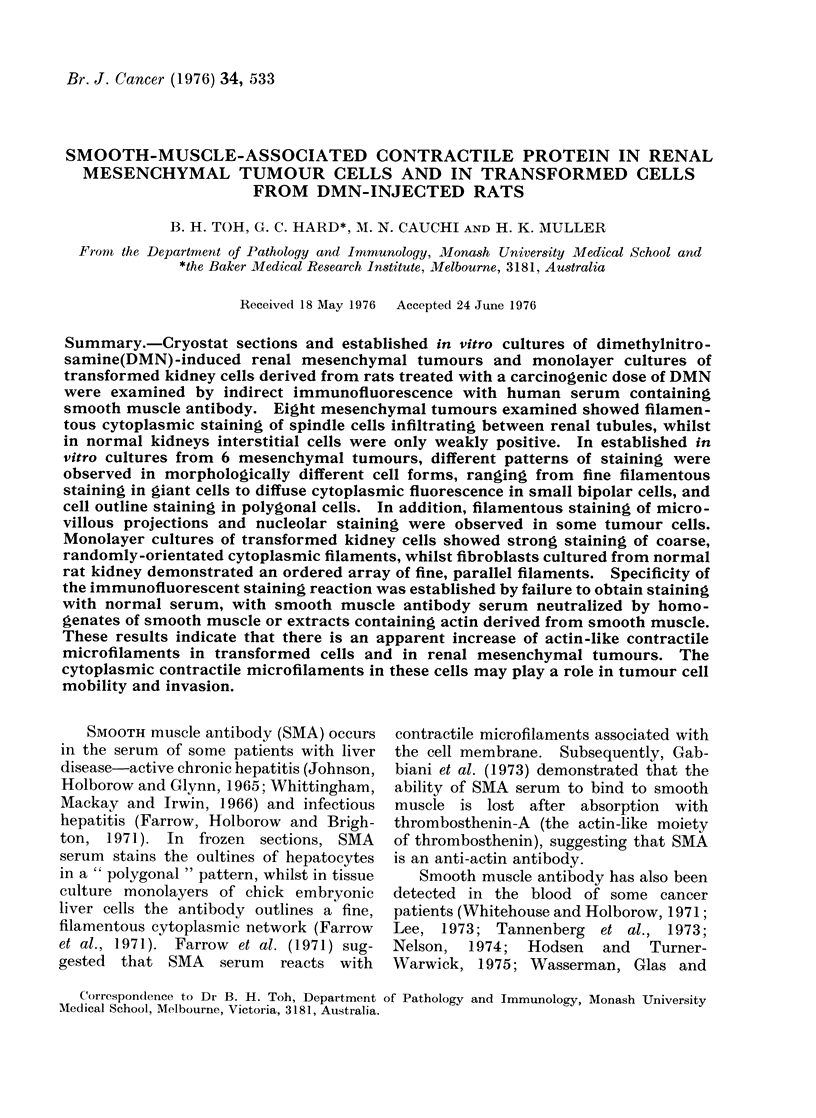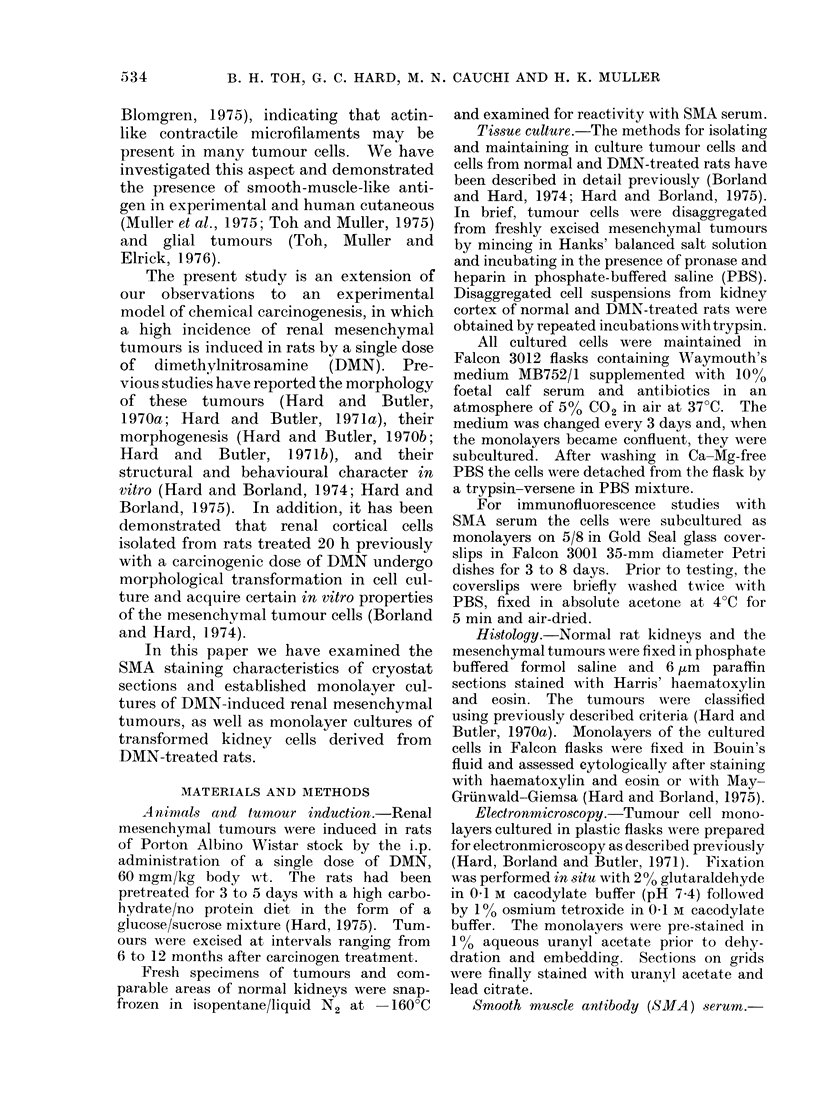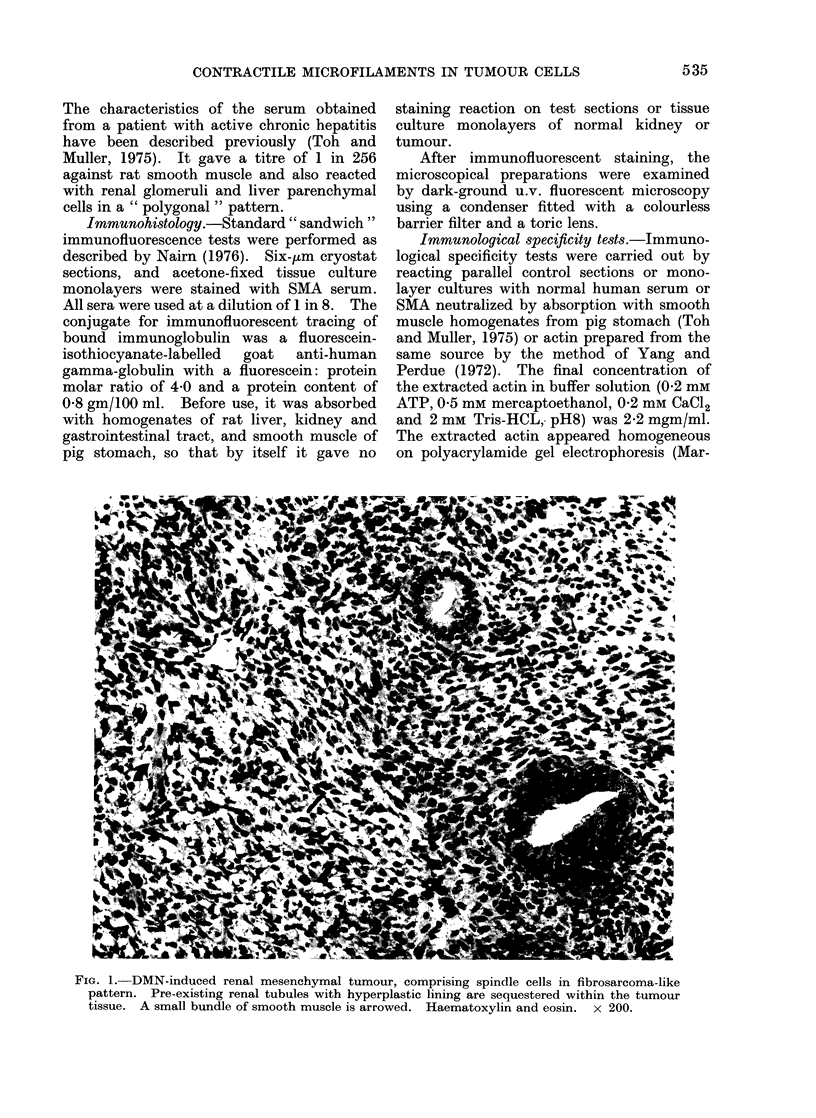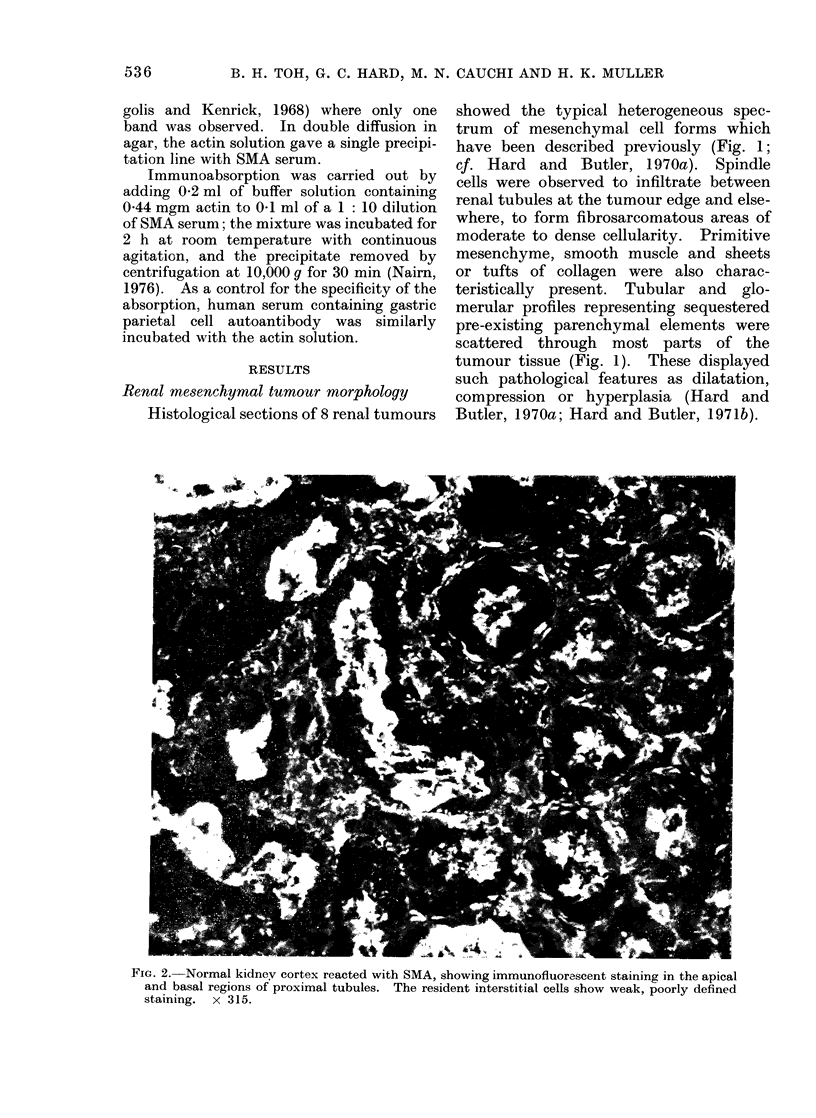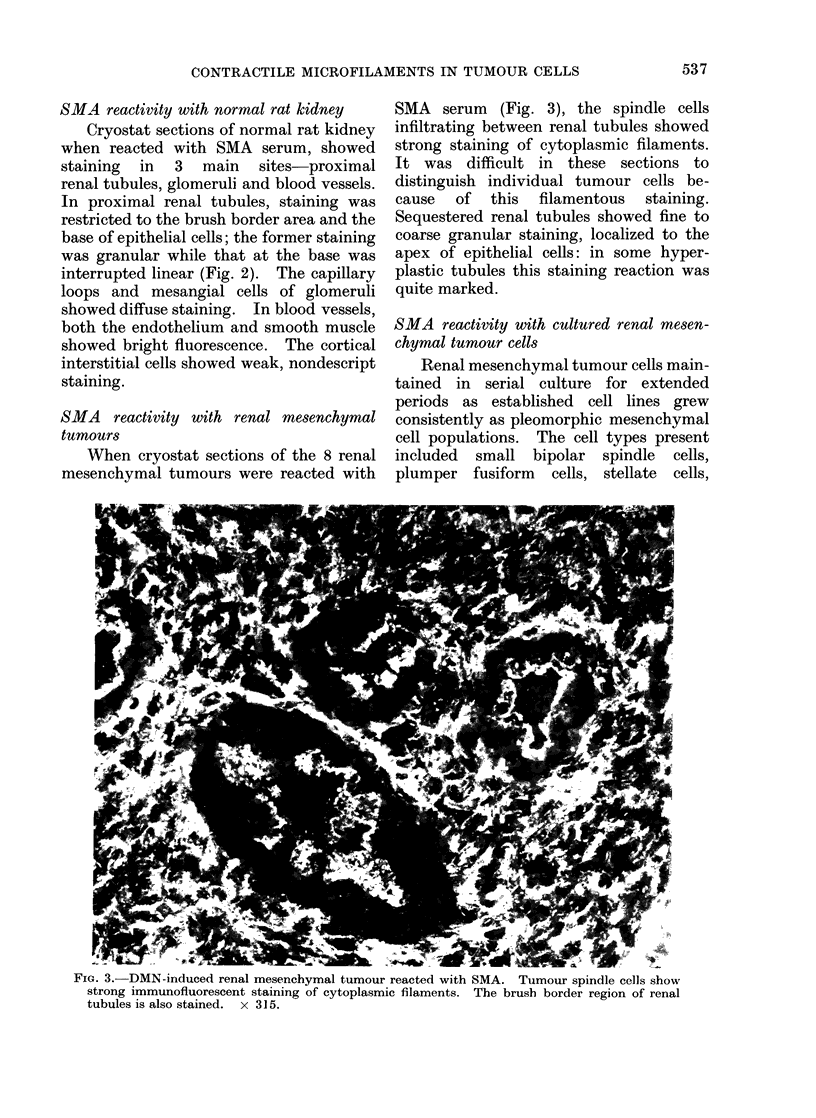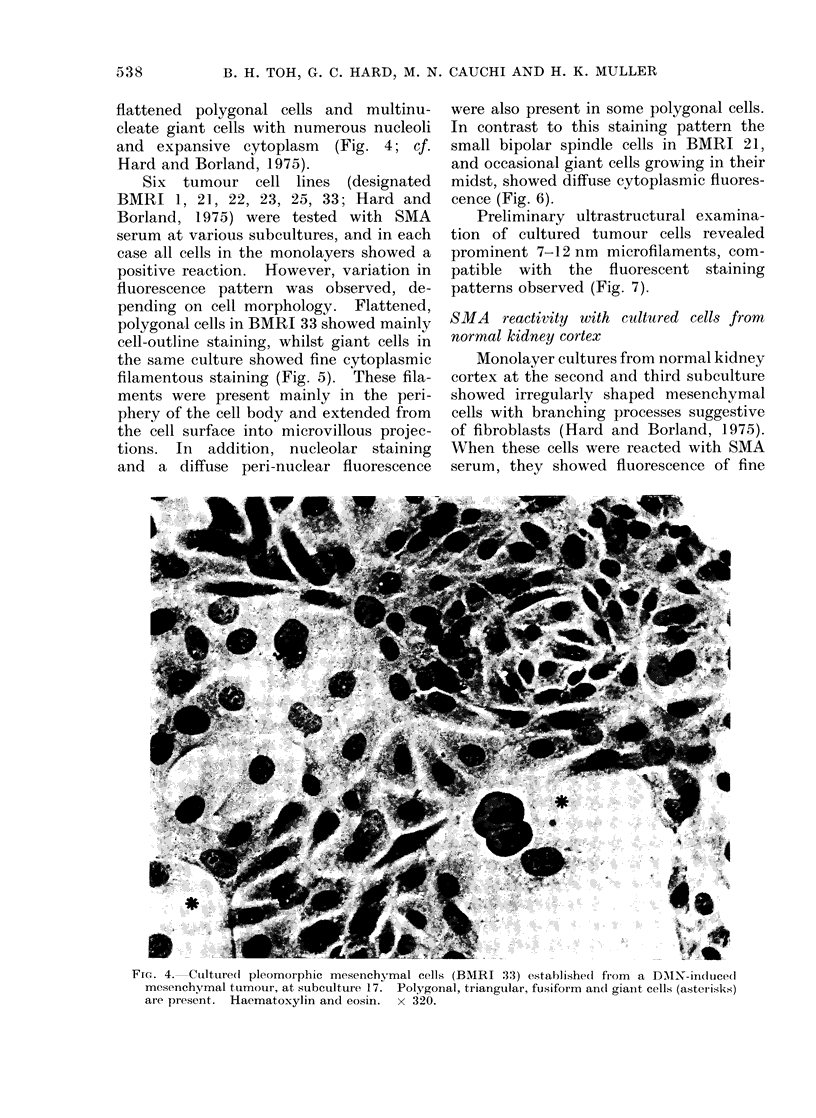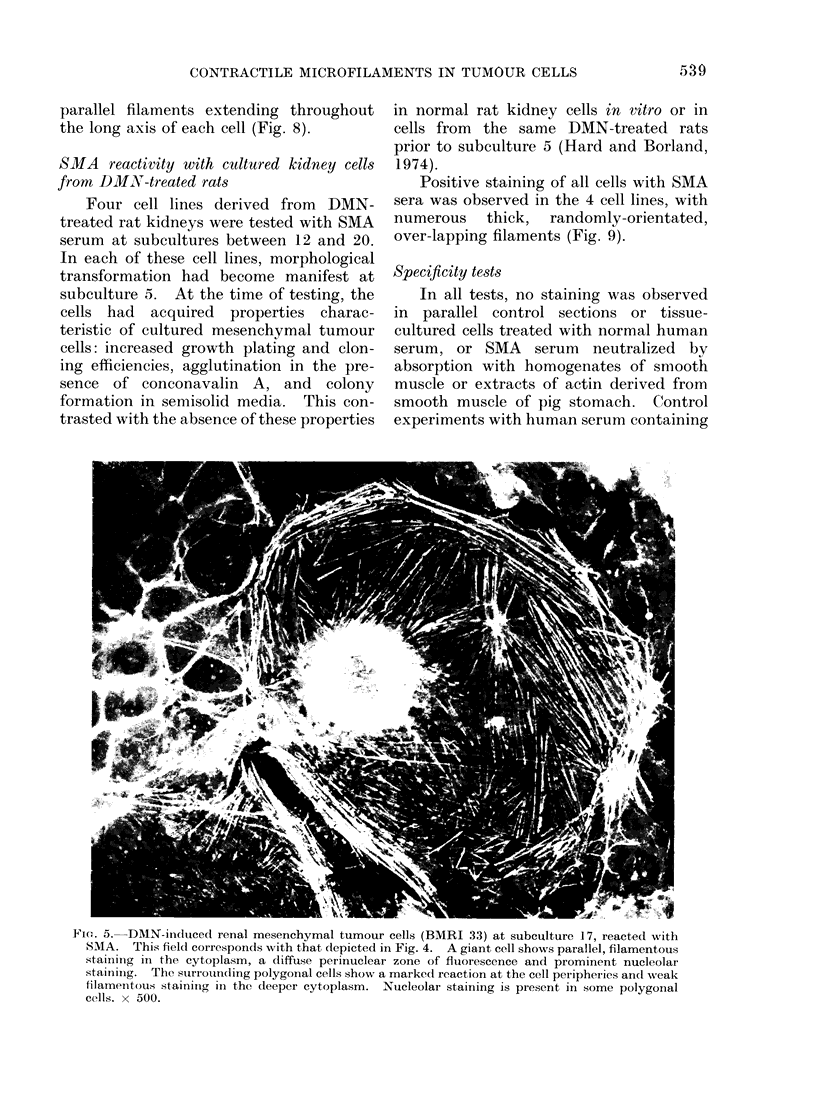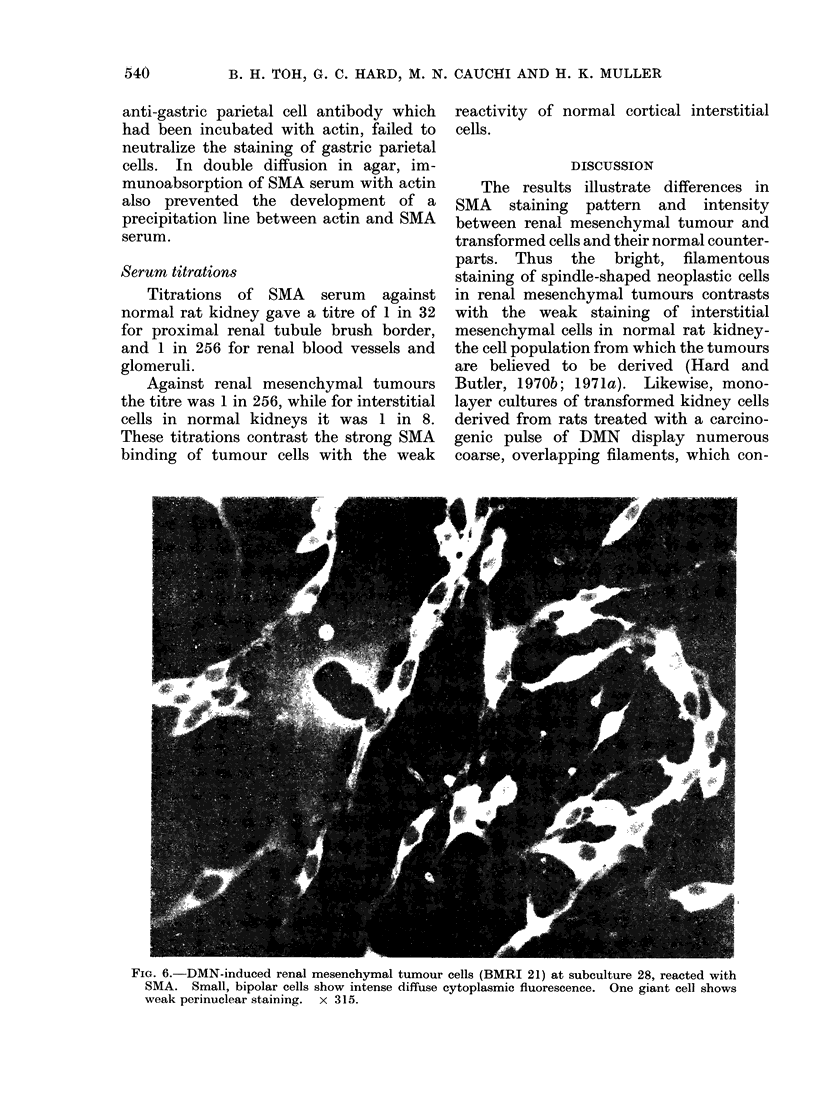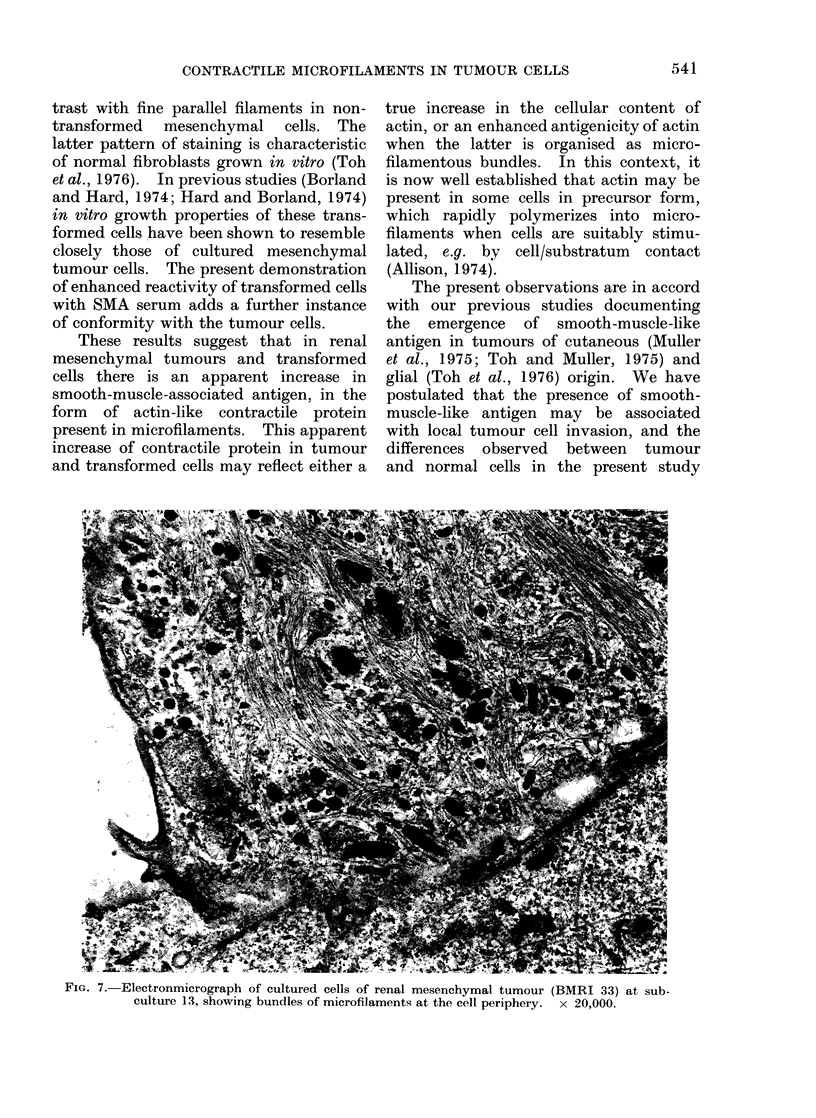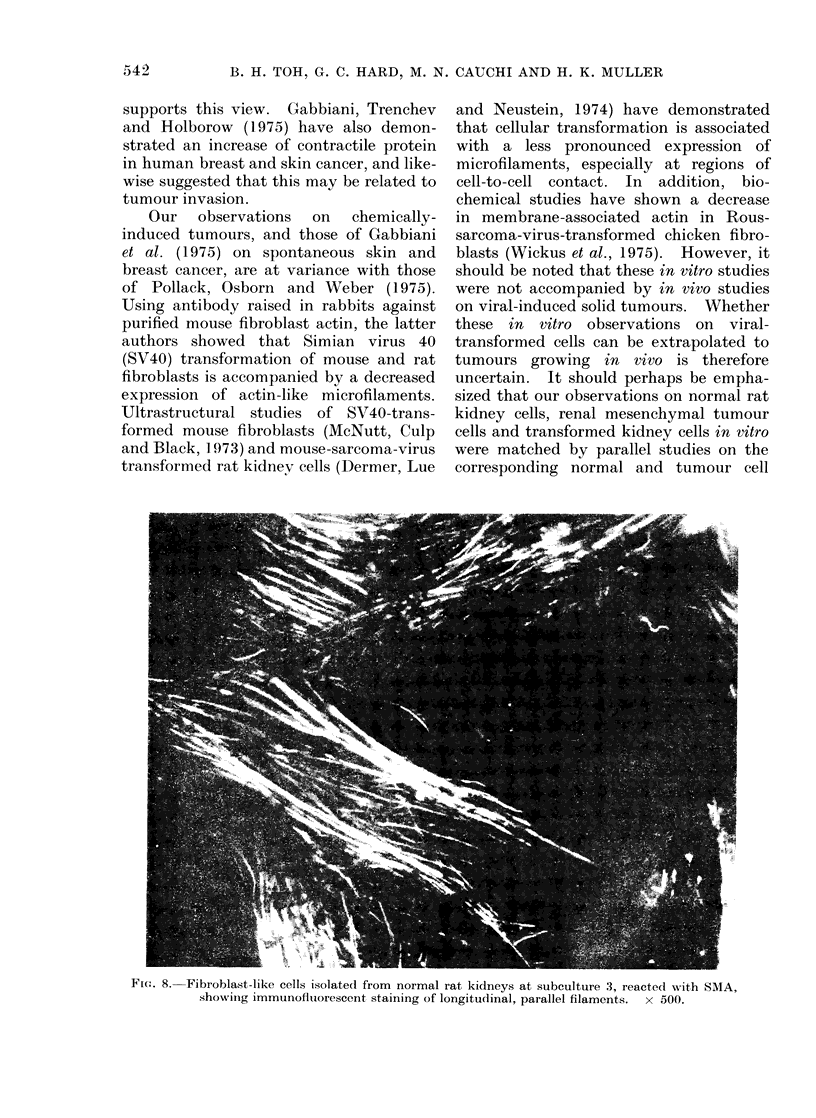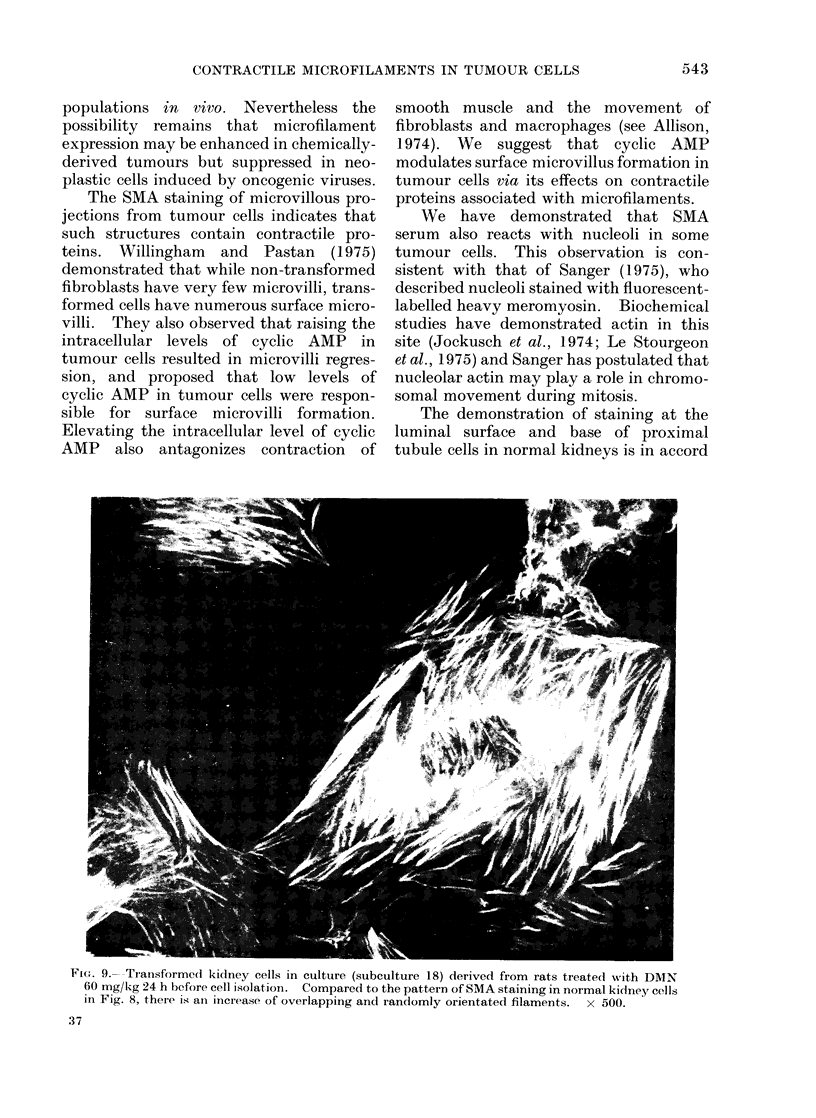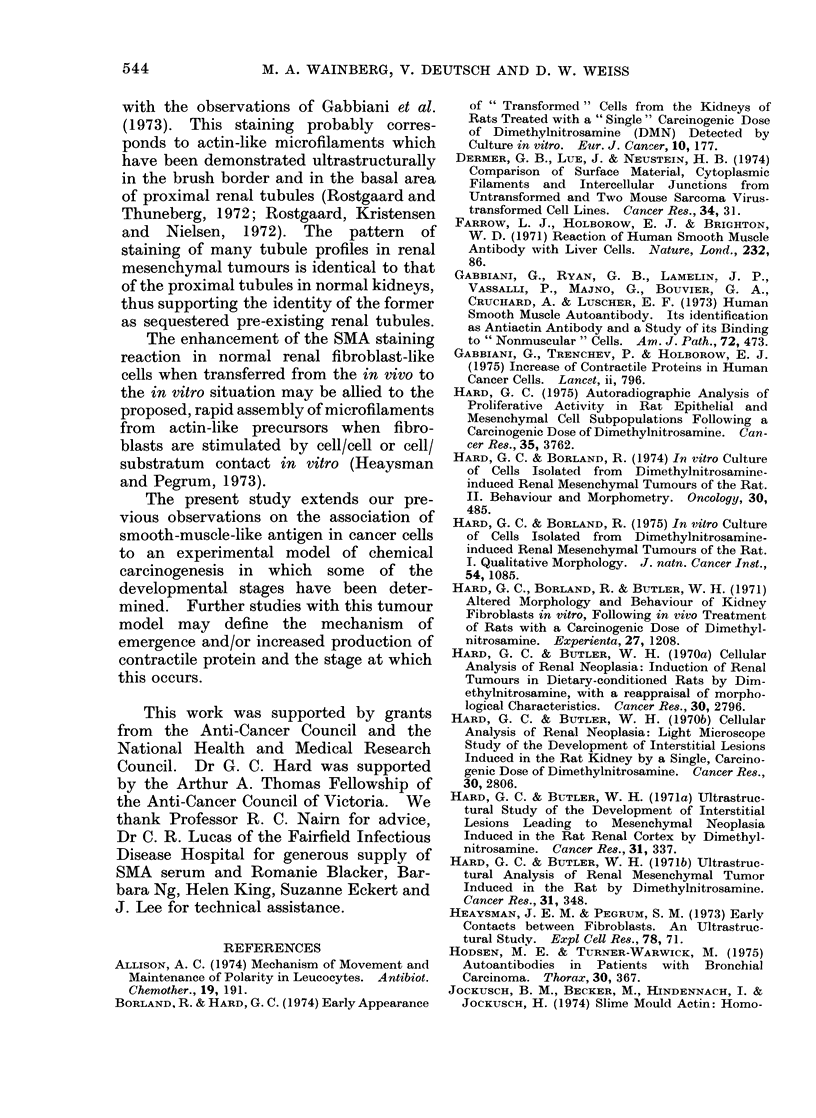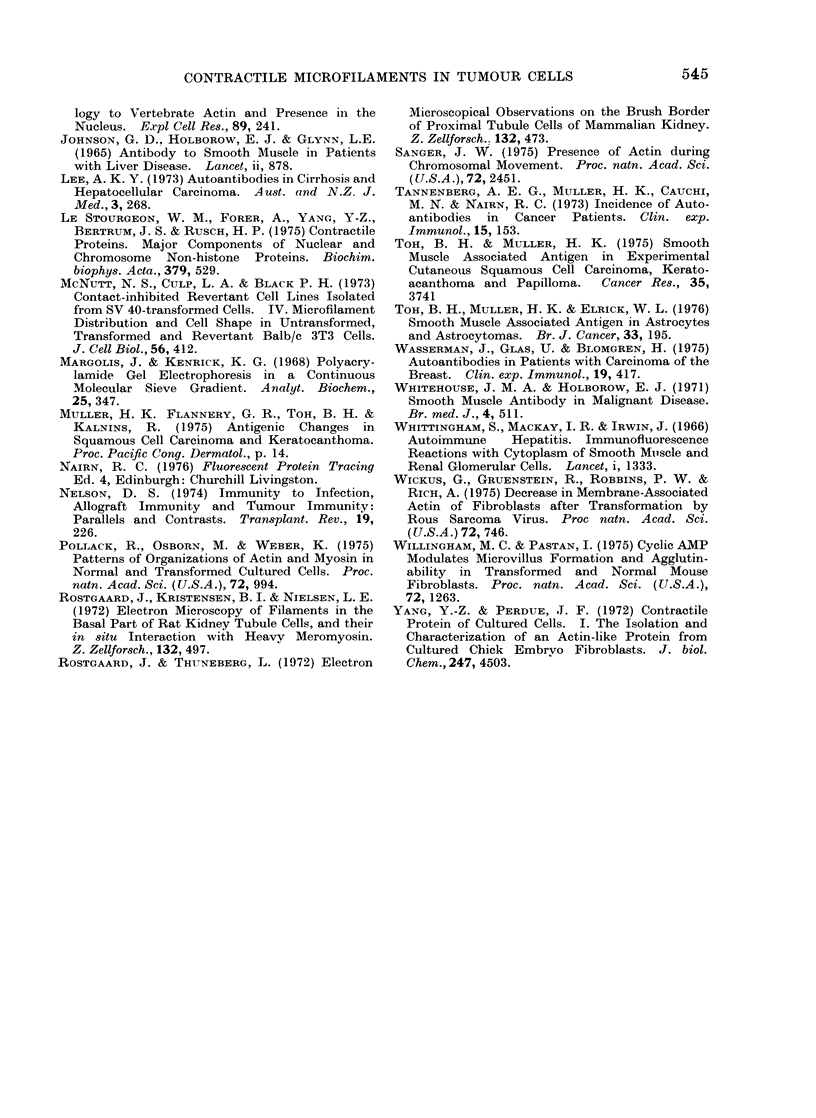# Smooth-muscle-associated contractile protein in renal mesenchymal tumour cells and in transformed cells from DMN-injected rats.

**DOI:** 10.1038/bjc.1976.208

**Published:** 1976-11

**Authors:** B. H. Toh, G. C. Hard, M. N. Cauchi, H. K. Muller

## Abstract

**Images:**


*Br. J. Cancer* (1976) **34**, 533

### SMOOTH-MUSCLE-ASSOCIATED CONTRACTILE PROTEIN IN RENAL MESENCHYMAL TUMOUR CELLS AND IN TRANSFORMED CELLS FROM DMN-INJECTED RATS

B. H. TOH, G. C. HARD*, M. N. CAUCHI AND H. K. MULLER

*From the Department of Pathology and Immunology, Monash University Medical School and *the Baker Medical Research Institute, Melbourne, 3181, Australia*

Received 18 May 1976 Accepted 24 June 1976

**Summary.—Cryostat sections and established *in vitro* cultures of dimethylnitrosamine(DMN)-induced renal mesenchymal tumours and monolayer cultures of transformed kidney cells derived from rats treated with a carcinogenic dose of DMN were examined by indirect immunofluorescence with human serum containing smooth muscle antibody. Eight mesenchymal tumours examined showed filamentous cytoplasmic staining of spindle cells infiltrating between renal tubules, whilst in normal kidneys interstitial cells were only weakly positive. In established *in vitro* cultures from 6 mesenchymal tumours, different patterns of staining were observed in morphologically different cell forms, ranging from fine filamentous staining in giant cells to diffuse cytoplasmic fluorescence in small bipolar cells, and cell outline staining in polygonal cells. In addition, filamentous staining of microvillous projections and nucleolar staining were observed in some tumour cells. Monolayer cultures of transformed kidney cells showed strong staining of coarse, randomly-orientated cytoplasmic filaments, whilst fibroblasts cultured from normal rat kidney demonstrated an ordered array of fine, parallel filaments. Specificity of the immunofluorescent staining reaction was established by failure to obtain staining with normal serum, with smooth muscle antibody serum neutralized by homogenates of smooth muscle or extracts containing actin derived from smooth muscle. These results indicate that there is an apparent increase of actin-like contractile microfilaments in transformed cells and in renal mesenchymal tumours. The cytoplasmic contractile microfilaments in these cells may play a role in tumour cell mobility and invasion.**

Smooth muscle antibody (SMA) occurs in the serum of some patients with liver disease—active chronic hepatitis (Johnson, Holborow and Glynn, 1965; Whittingham, Mackay and Irwin, 1966) and infectious hepatitis (Farrow, Holborow and Brighton, 1971). In frozen sections, SMA serum stains the oultines of hepatocytes in a "polygonal" pattern, whilst in tissue culture monolayers of chick embryonic liver cells the antibody outlines a fine, filamentous cytoplasmic network (Farrow *et al.*, 1971). Farrow *et al.* (1971) suggested that SMA serum reacts with contractile microfilaments associated with the cell membrane. Subsequently, Gabbiani *et al.* (1973) demonstrated that the ability of SMA serum to bind to smooth muscle is lost after absorption with thrombosthenin-A (the actin-like moiety of thrombosthenin), suggesting that SMA is an anti-actin antibody.

Smooth muscle antibody has also been detected in the blood of some cancer patients (Whitehouse and Holborow, 1971; Lee, 1973; Tannenberg *et al.*, 1973; Nelson, 1974; Hodsen and Turner-Warwick, 1975; Wasserman, Glas and

Correspondence to Dr B. H. Toh, Department of Pathology and Immunology, Monash University Medical School, Melbourne, Victoria, 3181, Australia.

Blomgren, 1975), indicating that actin-like contractile microfilaments may be present in many tumour cells. We have investigated this aspect and demonstrated the presence of smooth-muscle-like antigen in experimental and human cutaneous (Muller *et al.*, 1975; Toh and Muller, 1975) and glial tumours (Toh, Muller and Elrick, 1976).

The present study is an extension of our observations to an experimental model of chemical carcinogenesis, in which a high incidence of renal mesenchymal tumours is induced in rats by a single dose of dimethylnitrosamine (DMN). Previous studies have reported the morphology of these tumours (Hard and Butler, 1970*a*; Hard and Butler, 1971*a*), their morphogenesis (Hard and Butler, 1970*b*; Hard and Butler, 1971*b*), and their structural and behavioural character *in vitro* (Hard and Borland, 1974; Hard and Borland, 1975). In addition, it has been demonstrated that renal cortical cells isolated from rats treated 20 h previously with a carcinogenic dose of DMN undergo morphological transformation in cell culture and acquire certain *in vitro* properties of the mesenchymal tumour cells (Borland and Hard, 1974).

In this paper we have examined the SMA staining characteristics of cryostat sections and established monolayer cultures of DMN-induced renal mesenchymal tumours, as well as monolayer cultures of transformed kidney cells derived from DMN-treated rats.

### MATERIALS AND METHODS

*Animals and tumour induction.*—Renal mesenchymal tumours were induced in rats of Porton Albino Wistar stock by the i.p. administration of a single dose of DMN, 60 mgm/kg body wt. The rats had been pretreated for 3 to 5 days with a high carbohydrate/no protein diet in the form of a glucose/sucrose mixture (Hard, 1975). Tumours were excised at intervals ranging from 6 to 12 months after carcinogen treatment.

Fresh specimens of tumours and comparable areas of normal kidneys were snap-frozen in isopentane/liquid $N_2$ at $-160°C$ and examined for reactivity with SMA serum.

*Tissue culture.*—The methods for isolating and maintaining in culture tumour cells and cells from normal and DMN-treated rats have been described in detail previously (Borland and Hard, 1974; Hard and Borland, 1975). In brief, tumour cells were disaggregated from freshly excised mesenchymal tumours by mincing in Hanks' balanced salt solution and incubating in the presence of pronase and heparin in phosphate-buffered saline (PBS). Disaggregated cell suspensions from kidney cortex of normal and DMN-treated rats were obtained by repeated incubations with trypsin.

All cultured cells were maintained in Falcon 3012 flasks containing Waymouth's medium MB752/1 supplemented with 10% foetal calf serum and antibiotics in an atmosphere of 5% $CO_2$ in air at 37°C. The medium was changed every 3 days and, when the monolayers became confluent, they were subcultured. After washing in Ca–Mg-free PBS the cells were detached from the flask by a trypsin–versene in PBS mixture.

For immunofluorescence studies with SMA serum the cells were subcultured as monolayers on 5/8 in Gold Seal glass coverslips in Falcon 3001 35-mm diameter Petri dishes for 3 to 8 days. Prior to testing, the coverslips were briefly washed twice with PBS, fixed in absolute acetone at 4°C for 5 min and air-dried.

*Histology.*—Normal rat kidneys and the mesenchymal tumours were fixed in phosphate buffered formol saline and 6 $\mu$m paraffin sections stained with Harris' haematoxylin and eosin. The tumours were classified using previously described criteria (Hard and Butler, 1970*a*). Monolayers of the cultured cells in Falcon flasks were fixed in Bouin's fluid and assessed cytologically after staining with haematoxylin and eosin or with May–Grünwald–Giemsa (Hard and Borland, 1975).

*Electronmicroscopy.*—Tumour cell monolayers cultured in plastic flasks were prepared for electronmicroscopy as described previously (Hard, Borland and Butler, 1971). Fixation was performed *in situ* with 2% glutaraldehyde in 0·1 M cacodylate buffer (pH 7·4) followed by 1% osmium tetroxide in 0·1 M cacodylate buffer. The monolayers were pre-stained in 1% aqueous uranyl acetate prior to dehydration and embedding. Sections on grids were finally stained with uranyl acetate and lead citrate.

*Smooth muscle antibody* (*SMA*) *serum.*—

The characteristics of the serum obtained from a patient with active chronic hepatitis have been described previously (Toh and Muller, 1975). It gave a titre of 1 in 256 against rat smooth muscle and also reacted with renal glomeruli and liver parenchymal cells in a " polygonal " pattern.

*Immunohistology.*—Standard " sandwich " immunofluorescence tests were performed as described by Nairn (1976). Six-$\mu$m cryostat sections, and acetone-fixed tissue culture monolayers were stained with SMA serum. All sera were used at a dilution of 1 in 8. The conjugate for immunofluorescent tracing of bound immunoglobulin was a fluorescein-isothiocyanate-labelled goat anti-human gamma-globulin with a fluorescein: protein molar ratio of 4·0 and a protein content of 0·8 gm/100 ml. Before use, it was absorbed with homogenates of rat liver, kidney and gastrointestinal tract, and smooth muscle of pig stomach, so that by itself it gave no staining reaction on test sections or tissue culture monolayers of normal kidney or tumour.

After immunofluorescent staining, the microscopical preparations were examined by dark-ground u.v. fluorescent microscopy using a condenser fitted with a colourless barrier filter and a toric lens.

*Immunological specificity tests.*—Immunological specificity tests were carried out by reacting parallel control sections or monolayer cultures with normal human serum or SMA neutralized by absorption with smooth muscle homogenates from pig stomach (Toh and Muller, 1975) or actin prepared from the same source by the method of Yang and Perdue (1972). The final concentration of the extracted actin in buffer solution (0·2 mM ATP, 0·5 mM mercaptoethanol, 0·2 mM $CaCl_2$ and 2 mM Tris-HCL, pH8) was 2·2 mgm/ml. The extracted actin appeared homogeneous on polyacrylamide gel electrophoresis (Mar-

FIG. 1.—DMN-induced renal mesenchymal tumour, comprising spindle cells in fibrosarcoma-like pattern. Pre-existing renal tubules with hyperplastic lining are sequestered within the tumour tissue. A small bundle of smooth muscle is arrowed. Haematoxylin and eosin. × 200.

golis and Kenrick, 1968) where only one band was observed. In double diffusion in agar, the actin solution gave a single precipitation line with SMA serum.

Immunoabsorption was carried out by adding 0·2 ml of buffer solution containing 0·44 mgm actin to 0·1 ml of a 1 : 10 dilution of SMA serum; the mixture was incubated for 2 h at room temperature with continuous agitation, and the precipitate removed by centrifugation at 10,000 *g* for 30 min (Nairn, 1976). As a control for the specificity of the absorption, human serum containing gastric parietal cell autoantibody was similarly incubated with the actin solution.

### RESULTS

### *Renal mesenchymal tumour morphology*

Histological sections of 8 renal tumours showed the typical heterogeneous spectrum of mesenchymal cell forms which have been described previously (Fig. 1; *cf.* Hard and Butler, 1970*a*). Spindle cells were observed to infiltrate between renal tubules at the tumour edge and elsewhere, to form fibrosarcomatous areas of moderate to dense cellularity. Primitive mesenchyme, smooth muscle and sheets or tufts of collagen were also characteristically present. Tubular and glomerular profiles representing sequestered pre-existing parenchymal elements were scattered through most parts of the tumour tissue (Fig. 1). These displayed such pathological features as dilatation, compression or hyperplasia (Hard and Butler, 1970*a*; Hard and Butler, 1971*b*).

FIG. 2.—Normal kidney cortex reacted with SMA, showing immunofluorescent staining in the apical and basal regions of proximal tubules. The resident interstitial cells show weak, poorly defined staining. × 315.

*SMA reactivity with normal rat kidney*

Cryostat sections of normal rat kidney when reacted with SMA serum, showed staining in 3 main sites—proximal renal tubules, glomeruli and blood vessels. In proximal renal tubules, staining was restricted to the brush border area and the base of epithelial cells; the former staining was granular while that at the base was interrupted linear (Fig. 2). The capillary loops and mesangial cells of glomeruli showed diffuse staining. In blood vessels, both the endothelium and smooth muscle showed bright fluorescence. The cortical interstitial cells showed weak, nondescript staining.

*SMA reactivity with renal mesenchymal tumours*

When cryostat sections of the 8 renal mesenchymal tumours were reacted with SMA serum (Fig. 3), the spindle cells infiltrating between renal tubules showed strong staining of cytoplasmic filaments. It was difficult in these sections to distinguish individual tumour cells because of this filamentous staining. Sequestered renal tubules showed fine to coarse granular staining, localized to the apex of epithelial cells: in some hyperplastic tubules this staining reaction was quite marked.

*SMA reactivity with cultured renal mesenchymal tumour cells*

Renal mesenchymal tumour cells maintained in serial culture for extended periods as established cell lines grew consistently as pleomorphic mesenchymal cell populations. The cell types present included small bipolar spindle cells, plumper fusiform cells, stellate cells,

FIG. 3.—DMN-induced renal mesenchymal tumour reacted with SMA. Tumour spindle cells show strong immunofluorescent staining of cytoplasmic filaments. The brush border region of renal tubules is also stained. × 315.

flattened polygonal cells and multinucleate giant cells with numerous nucleoli and expansive cytoplasm (Fig. 4; *cf.* Hard and Borland, 1975).

Six tumour cell lines (designated BMRI 1, 21, 22, 23, 25, 33; Hard and Borland, 1975) were tested with SMA serum at various subcultures, and in each case all cells in the monolayers showed a positive reaction. However, variation in fluorescence pattern was observed, depending on cell morphology. Flattened, polygonal cells in BMRI 33 showed mainly cell-outline staining, whilst giant cells in the same culture showed fine cytoplasmic filamentous staining (Fig. 5). These filaments were present mainly in the periphery of the cell body and extended from the cell surface into microvillous projections. In addition, nucleolar staining and a diffuse peri-nuclear fluorescence were also present in some polygonal cells. In contrast to this staining pattern the small bipolar spindle cells in BMRI 21, and occasional giant cells growing in their midst, showed diffuse cytoplasmic fluorescence (Fig. 6).

Preliminary ultrastructural examination of cultured tumour cells revealed prominent 7–12 nm microfilaments, compatible with the fluorescent staining patterns observed (Fig. 7).

*SMA reactivity with cultured cells from normal kidney cortex*

Monolayer cultures from normal kidney cortex at the second and third subculture showed irregularly shaped mesenchymal cells with branching processes suggestive of fibroblasts (Hard and Borland, 1975). When these cells were reacted with SMA serum, they showed fluorescence of fine

FIG. 4.—Cultured pleomorphic mesenchymal cells (BMRI 33) established from a DMN-induced mesenchymal tumour, at subculture 17. Polygonal, triangular, fusiform and giant cells (asterisks) are present. Haematoxylin and eosin. × 320.

parallel filaments extending throughout the long axis of each cell (Fig. 8).

*SMA reactivity with cultured kidney cells from DMN-treated rats*

Four cell lines derived from DMN-treated rat kidneys were tested with SMA serum at subcultures between 12 and 20. In each of these cell lines, morphological transformation had become manifest at subculture 5. At the time of testing, the cells had acquired properties characteristic of cultured mesenchymal tumour cells: increased growth plating and cloning efficiencies, agglutination in the presence of conconavalin A, and colony formation in semisolid media. This contrasted with the absence of these properties in normal rat kidney cells *in vitro* or in cells from the same DMN-treated rats prior to subculture 5 (Hard and Borland, 1974).

Positive staining of all cells with SMA sera was observed in the 4 cell lines, with numerous thick, randomly-orientated, over-lapping filaments (Fig. 9).

*Specificity tests*

In all tests, no staining was observed in parallel control sections or tissue-cultured cells treated with normal human serum, or SMA serum neutralized by absorption with homogenates of smooth muscle or extracts of actin derived from smooth muscle of pig stomach. Control experiments with human serum containing

FIG. 5.—DMN-induced renal mesenchymal tumour cells (BMRI 33) at subculture 17, reacted with SMA. This field corresponds with that depicted in Fig. 4. A giant cell shows parallel, filamentous staining in the cytoplasm, a diffuse perinuclear zone of fluorescence and prominent nucleolar staining. The surrounding polygonal cells show a marked reaction at the cell peripheries and weak filamentous staining in the deeper cytoplasm. Nucleolar staining is present in some polygonal cells. × 500.

anti-gastric parietal cell antibody which had been incubated with actin, failed to neutralize the staining of gastric parietal cells. In double diffusion in agar, immunoabsorption of SMA serum with actin also prevented the development of a precipitation line between actin and SMA serum.

*Serum titrations*

Titrations of SMA serum against normal rat kidney gave a titre of 1 in 32 for proximal renal tubule brush border, and 1 in 256 for renal blood vessels and glomeruli.

Against renal mesenchymal tumours the titre was 1 in 256, while for interstitial cells in normal kidneys it was 1 in 8. These titrations contrast the strong SMA binding of tumour cells with the weak reactivity of normal cortical interstitial cells.

DISCUSSION

The results illustrate differences in SMA staining pattern and intensity between renal mesenchymal tumour and transformed cells and their normal counterparts. Thus the bright, filamentous staining of spindle-shaped neoplastic cells in renal mesenchymal tumours contrasts with the weak staining of interstitial mesenchymal cells in normal rat kidney-the cell population from which the tumours are believed to be derived (Hard and Butler, 1970*b*; 1971*a*). Likewise, monolayer cultures of transformed kidney cells derived from rats treated with a carcinogenic pulse of DMN display numerous coarse, overlapping filaments, which con-

FIG. 6.—DMN-induced renal mesenchymal tumour cells (BMRI 21) at subculture 28, reacted with SMA. Small, bipolar cells show intense diffuse cytoplasmic fluorescence. One giant cell shows weak perinuclear staining. × 315.

trast with fine parallel filaments in non-transformed mesenchymal cells. The latter pattern of staining is characteristic of normal fibroblasts grown *in vitro* (Toh *et al.*, 1976). In previous studies (Borland and Hard, 1974; Hard and Borland, 1974) *in vitro* growth properties of these transformed cells have been shown to resemble closely those of cultured mesenchymal tumour cells. The present demonstration of enhanced reactivity of transformed cells with SMA serum adds a further instance of conformity with the tumour cells.

These results suggest that in renal mesenchymal tumours and transformed cells there is an apparent increase in smooth-muscle-associated antigen, in the form of actin-like contractile protein present in microfilaments. This apparent increase of contractile protein in tumour and transformed cells may reflect either a true increase in the cellular content of actin, or an enhanced antigenicity of actin when the latter is organised as microfilamentous bundles. In this context, it is now well established that actin may be present in some cells in precursor form, which rapidly polymerizes into microfilaments when cells are suitably stimulated, *e.g.* by cell/substratum contact (Allison, 1974).

The present observations are in accord with our previous studies documenting the emergence of smooth-muscle-like antigen in tumours of cutaneous (Muller *et al.*, 1975; Toh and Muller, 1975) and glial (Toh *et al.*, 1976) origin. We have postulated that the presence of smooth-muscle-like antigen may be associated with local tumour cell invasion, and the differences observed between tumour and normal cells in the present study

FIG. 7.—Electronmicrograph of cultured cells of renal mesenchymal tumour (BMRI 33) at subculture 13, showing bundles of microfilaments at the cell periphery. × 20,000.

supports this view. Gabbiani, Trenchev and Holborow (1975) have also demonstrated an increase of contractile protein in human breast and skin cancer, and likewise suggested that this may be related to tumour invasion.

Our observations on chemically-induced tumours, and those of Gabbiani *et al.* (1975) on spontaneous skin and breast cancer, are at variance with those of Pollack, Osborn and Weber (1975). Using antibody raised in rabbits against purified mouse fibroblast actin, the latter authors showed that Simian virus 40 (SV40) transformation of mouse and rat fibroblasts is accompanied by a decreased expression of actin-like microfilaments. Ultrastructural studies of SV40-transformed mouse fibroblasts (McNutt, Culp and Black, 1973) and mouse-sarcoma-virus transformed rat kidney cells (Dermer, Lue and Neustein, 1974) have demonstrated that cellular transformation is associated with a less pronounced expression of microfilaments, especially at regions of cell-to-cell contact. In addition, biochemical studies have shown a decrease in membrane-associated actin in Rous-sarcoma-virus-transformed chicken fibroblasts (Wickus *et al.*, 1975). However, it should be noted that these *in vitro* studies were not accompanied by *in vivo* studies on viral-induced solid tumours. Whether these *in vitro* observations on viral-transformed cells can be extrapolated to tumours growing *in vivo* is therefore uncertain. It should perhaps be emphasized that our observations on normal rat kidney cells, renal mesenchymal tumour cells and transformed kidney cells *in vitro* were matched by parallel studies on the corresponding normal and tumour cell

FIG. 8.—Fibroblast-like cells isolated from normal rat kidneys at subculture 3, reacted with SMA, showing immunofluorescent staining of longitudinal, parallel filaments. × 500.

populations *in vivo*. Nevertheless the possibility remains that microfilament expression may be enhanced in chemically-derived tumours but suppressed in neoplastic cells induced by oncogenic viruses.

The SMA staining of microvillous projections from tumour cells indicates that such structures contain contractile proteins. Willingham and Pastan (1975) demonstrated that while non-transformed fibroblasts have very few microvilli, transformed cells have numerous surface microvilli. They also observed that raising the intracellular levels of cyclic AMP in tumour cells resulted in microvilli regression, and proposed that low levels of cyclic AMP in tumour cells were responsible for surface microvilli formation. Elevating the intracellular level of cyclic AMP also antagonizes contraction of smooth muscle and the movement of fibroblasts and macrophages (see Allison, 1974). We suggest that cyclic AMP modulates surface microvillus formation in tumour cells *via* its effects on contractile proteins associated with microfilaments.

We have demonstrated that SMA serum also reacts with nucleoli in some tumour cells. This observation is consistent with that of Sanger (1975), who described nucleoli stained with fluorescent-labelled heavy meromyosin. Biochemical studies have demonstrated actin in this site (Jockusch *et al.*, 1974; Le Stourgeon *et al.*, 1975) and Sanger has postulated that nucleolar actin may play a role in chromosomal movement during mitosis.

The demonstration of staining at the luminal surface and base of proximal tubule cells in normal kidneys is in accord

FIG. 9.—Transformed kidney cells in culture (subculture 18) derived from rats treated with DMN 60 mg/kg 24 h before cell isolation. Compared to the pattern of SMA staining in normal kidney cells in Fig. 8, there is an increase of overlapping and randomly orientated filaments. × 500.

with the observations of Gabbiani *et al.* (1973). This staining probably corresponds to actin-like microfilaments which have been demonstrated ultrastructurally in the brush border and in the basal area of proximal renal tubules (Rostgaard and Thuneberg, 1972; Rostgaard, Kristensen and Nielsen, 1972). The pattern of staining of many tubule profiles in renal mesenchymal tumours is identical to that of the proximal tubules in normal kidneys, thus supporting the identity of the former as sequestered pre-existing renal tubules.

The enhancement of the SMA staining reaction in normal renal fibroblast-like cells when transferred from the *in vivo* to the *in vitro* situation may be allied to the proposed, rapid assembly of microfilaments from actin-like precursors when fibroblasts are stimulated by cell/cell or cell/substratum contact *in vitro* (Heaysman and Pegrum, 1973).

The present study extends our previous observations on the association of smooth-muscle-like antigen in cancer cells to an experimental model of chemical carcinogenesis in which some of the developmental stages have been determined. Further studies with this tumour model may define the mechanism of emergence and/or increased production of contractile protein and the stage at which this occurs.

This work was supported by grants from the Anti-Cancer Council and the National Health and Medical Research Council. Dr G. C. Hard was supported by the Arthur A. Thomas Fellowship of the Anti-Cancer Council of Victoria. We thank Professor R. C. Nairn for advice, Dr C. R. Lucas of the Fairfield Infectious Disease Hospital for generous supply of SMA serum and Romanie Blacker, Barbara Ng, Helen King, Suzanne Eckert and J. Lee for technical assistance.

REFERENCES

ALLISON, A. C. (1974) Mechanism of Movement and Maintenance of Polarity in Leucocytes. *Antibiot. Chemother.*, **19**, 191.

BORLAND, R. & HARD, G. C. (1974) Early Appearance of " Transformed " Cells from the Kidneys of Rats Treated with a " Single " Carcinogenic Dose of Dimethylnitrosamine (DMN) Detected by Culture *in vitro*. *Eur. J. Cancer*, **10**, 177.

DERMER, G. B., LUE, J. & NEUSTEIN, H. B. (1974) Comparison of Surface Material, Cytoplasmic Filaments and Intercellular Junctions from Untransformed and Two Mouse Sarcoma Virus-transformed Cell Lines. *Cancer Res.*, **34**, 31.

FARROW, L. J., HOLBOROW, E. J. & BRIGHTON, W. D. (1971) Reaction of Human Smooth Muscle Antibody with Liver Cells. *Nature, Lond.*, **232**, 86.

GABBIANI, G., RYAN, G. B., LAMELIN, J. P., VASSALLI, P., MAJNO, G., BOUVIER, G. A., CRUCHARD, A. & LUSCHER, E. F. (1973) Human Smooth Muscle Autoantibody. Its identification as Antiactin Antibody and a Study of its Binding to " Nonmuscular " Cells. *Am. J. Path.*, **72**, 473.

GABBIANI, G., TRENCHEV, P. & HOLBOROW, E. J. (1975) Increase of Contractile Proteins in Human Cancer Cells. *Lancet*, ii, 796.

HARD, G. C. (1975) Autoradiographic Analysis of Proliferative Activity in Rat Epithelial and Mesenchymal Cell Subpopulations Following a Carcinogenic Dose of Dimethylnitrosamine. *Cancer Res.*, **35**, 3762.

HARD, G. C. & BORLAND, R. (1974) *In vitro* Culture of Cells Isolated from Dimethylnitrosamine-induced Renal Mesenchymal Tumours of the Rat. II. Behaviour and Morphometry. *Oncology*, **30**, 485.

HARD, G. C. & BORLAND, R. (1975) *In vitro* Culture of Cells Isolated from Dimethylnitrosamine-induced Renal Mesenchymal Tumours of the Rat. I. Qualitative Morphology. *J. natn. Cancer Inst.*, **54**, 1085.

HARD, G. C., BORLAND, R. & BUTLER, W. H. (1971) Altered Morphology and Behaviour of Kidney Fibroblasts *in vitro*, Following *in vivo* Treatment of Rats with a Carcinogenic Dose of Dimethylnitrosamine. *Experienta*, **27**, 1208.

HARD, G. C. & BUTLER, W. H. (1970*a*) Cellular Analysis of Renal Neoplasia: Induction of Renal Tumours in Dietary-conditioned Rats by Dimethylnitrosamine, with a reappraisal of morphological Characteristics. *Cancer Res.*, **30**, 2796.

HARD, G. C. & BUTLER, W. H. (1970*b*) Cellular Analysis of Renal Neoplasia: Light Microscope Study of the Development of Interstitial Lesions Induced in the Rat Kidney by a Single, Carcinogenic Dose of Dimethylnitrosamine. *Cancer Res.*, **30**, 2806.

HARD, G. C. & BUTLER, W. H. (1971*a*) Ultrastructural Study of the Development of Interstitial Lesions Leading to Mesenchymal Neoplasia Induced in the Rat Renal Cortex by Dimethylnitrosamine. *Cancer Res.*, **31**, 337.

HARD, G. C. & BUTLER, W. H. (1971*b*) Ultrastructural Analysis of Renal Mesenchymal Tumor Induced in the Rat by Dimethylnitrosamine. *Cancer Res.*, **31**, 348.

HEAYSMAN, J. E. M. & PEGRUM, S. M. (1973) Early Contacts between Fibroblasts. An Ultrastructural Study. *Expl Cell Res.*, **78**, 71.

HODSEN, M. E. & TURNER-WARWICK, M. (1975) Autoantibodies in Patients with Bronchial Carcinoma. *Thorax*, **30**, 367.

JOCKUSCH, B. M., BECKER, M., HINDENNACH, I. & JOCKUSCH, H. (1974) Slime Mould Actin: Homology to Vertebrate Actin and Presence in the Nucleus. *Expl Cell Res.*, **89**, 241.

JOHNSON, G. D., HOLBOROW, E. J. & GLYNN, L.E. (1965) Antibody to Smooth Muscle in Patients with Liver Disease. *Lancet*, ii, 878.

LEE, A. K. Y. (1973) Autoantibodies in Cirrhosis and Hepatocellular Carcinoma. *Aust. and N.Z. J. Med.*, **3**, 268.

LE STOURGEON, W. M., FORER, A., YANG, Y-Z., BERTRUM, J. S. & RUSCH, H. P. (1975) Contractile Proteins. Major Components of Nuclear and Chromosome Non-histone Proteins. *Biochim. biophys. Acta.*, **379**, 529.

McNUTT, N. S., CULP, L. A. & BLACK P. H. (1973) Contact-inhibited Revertant Cell Lines Isolated from SV 40-transformed Cells. IV. Microfilament Distribution and Cell Shape in Untransformed, Transformed and Revertant Balb/c 3T3 Cells. *J. Cell Biol.*, **56**, 412.

MARGOLIS, J. & KENRICK, K. G. (1968) Polyacrylamide Gel Electrophoresis in a Continuous Molecular Sieve Gradient. *Analyt. Biochem.*, **25**, 347.

MULLER, H. K. FLANNERY, G. R., TOH, B. H. & KALNINS, R. (1975) Antigenic Changes in Squamous Cell Carcinoma and Keratocanthoma. *Proc. Pacific Cong. Dermatol.*, p. 14.

NAIRN, R. C. (1976) *Fluorescent Protein Tracing* Ed. 4, Edinburgh: Churchill Livingston.

NELSON, D. S. (1974) Immunity to Infection, Allograft Immunity and Tumour Immunity: Parallels and Contrasts. *Transplant. Rev.*, **19**, 226.

POLLACK, R., OSBORN, M. & WEBER, K. (1975) Patterns of Organizations of Actin and Myosin in Normal and Transformed Cultured Cells. *Proc. natn. Acad. Sci.* (*U.S.A.*), **72**, 994.

ROSTGAARD, J., KRISTENSEN, B. I. & NIELSEN, L. E. (1972) Electron Microscopy of Filaments in the Basal Part of Rat Kidney Tubule Cells, and their *in situ* Interaction with Heavy Meromyosin. *Z. Zellforsch.*, **132**, 497.

ROSTGAARD, J. & THUNEBERG, L. (1972) Electron Microscopical Observations on the Brush Border of Proximal Tubule Cells of Mammalian Kidney. *Z. Zellforsch.*, **132**, 473.

SANGER, J. W. (1975) Presence of Actin during Chromosomal Movement. *Proc. natn. Acad. Sci.* (*U.S.A.*), **72**, 2451.

TANNENBERG, A. E. G., MULLER, H. K., CAUCHI, M. N. & NAIRN, R. C. (1973) Incidence of Autoantibodies in Cancer Patients. *Clin. exp. Immunol.*, **15**, 153.

TOH, B. H. & MULLER, H. K. (1975) Smooth Muscle Associated Antigen in Experimental Cutaneous Squamous Cell Carcinoma, Keratoacanthoma and Papilloma. *Cancer Res.*, **35**, 3741

TOH, B. H., MULLER, H. K. & ELRICK, W. L. (1976) Smooth Muscle Associated Antigen in Astrocytes and Astrocytomas. *Br. J. Cancer*, **33**, 195.

WASSERMAN, J., GLAS, U. & BLOMGREN, H. (1975) Autoantibodies in Patients with Carcinoma of the Breast. *Clin. exp. Immunol.*, **19**, 417.

WHITEHOUSE, J. M. A. & HOLBOROW, E. J. (1971) Smooth Muscle Antibody in Malignant Disease. *Br. med. J.*, **4**, 511.

WHITTINGHAM, S., MACKAY, I. R. & IRWIN, J. (1966) Autoimmune Hepatitis. Immunofluorescence Reactions with Cytoplasm of Smooth Muscle and Renal Glomerular Cells. *Lancet*, i, 1333.

WICKUS, G., GRUENSTEIN, R., ROBBINS, P. W. & RICH, A. (1975) Decrease in Membrane-Associated Actin of Fibroblasts after Transformation by Rous Sarcoma Virus. *Proc natn. Acad. Sci.* (*U.S.A.*) **72**, 746.

WILLINGHAM, M. C. & PASTAN, I. (1975) Cyclic AMP Modulates Microvillus Formation and Agglutinability in Transformed and Normal Mouse Fibroblasts. *Proc. natn. Acad. Sci.* (*U.S.A.*), **72**, 1263.

YANG, Y.-Z. & PERDUE, J. F. (1972) Contractile Protein of Cultured Cells. I. The Isolation and Characterization of an Actin-like Protein from Cultured Chick Embryo Fibroblasts. *J. biol. Chem.*, **247**, 4503.